# Inactivation of *Escherichia coli* in an Orange Juice Beverage by Combined Ultrasonic and Microwave Treatment

**DOI:** 10.3390/foods12030666

**Published:** 2023-02-03

**Authors:** Ourdia-Nouara Kernou, Zahra Azzouz, Amine Belbahi, Kamelia Kerdouche, Ghania Kaanin-Boudraa, Akila Amir, Khodir Madani, Patricia Rijo

**Affiliations:** 1 Laboratoire de Biomathématiques, Biophysique, Biochimie, et Scientométrie (L3BS), Faculté des Sciences de la Nature et de la Vie, Université de Bejaia, Bejaia 06000, Algeria; 2 Laboratory of Applied Microbiology (LMA), Faculty of Natural and Life Sciences, University of Bejaia, Bejaia 06000, Algeria; 3 Department of Microbiology and Biochemistry, Faculty of Sciences, University of M’Sila, M’Sila 24000, Algeria; 4 Centre de Recherche en Technologie Agroalimentaire, Route de Targua-Ouzemour, Bejaia 06000, Algeria; 5 CBIOS-Centro de Investigação em Biociências e Tecnologias da Saúde, Universidade Lusófona, Campo Grande 376, 1749-028 Lisbon, Portugal; 6 Instituto de Investigação do Medicamento (iMed.ULisboa), Faculdade de Farmácia, Universidade de Lisboa,1649-003 Lisboa, Portugal

**Keywords:** *Escherichia coli* inactivation, optimization, ultrasound, microwave, RSM

## Abstract

The inactivation of Escherichia coli is one of the major issues in the food industry. The present study focuses on the application of a combined microwave-ultrasound system for the optimization of the inactivation of Escherichia coli ATCC 25922 in an orange juice drink. Using response surface methodology (RSM), trials were planned with a Box–Behnken Design (BBD) to maximize the impact of microwave power (A: 300–900 W), microwave treatment time (B: 15–35 s), and time of ultrasound (C: 10–30 min) on *E. coli* inactivation. Analysis of variance (ANOVA) was carried out and *E. coli* inactivation was expressed with a mathematical equation depending on the factors. The results showed that both the microwave treatment time and the time of ultrasound were effective as independent variables in eliminating the *E. coli* strain. However, the effect of these two variables, ultrasound and microwave exposure time, in combination was significantly greater than when examined separately. RSM modeling determined that optimal treatment conditions include 900 W microwave power, 33 s microwave treatment time, and 20 min time of ultrasound to achieve an 8-log reduction of *E. coli*, constituting total inactivation. The results of this study showed that ultrasound-microwave treatment is a potential alternative processing method for an orange juice beverage.

## 1. Introduction

Fruit juice is a medium rich in complex nutrients, which can be a favorable environment for the development of pathogens of food origin. Among these agents are alteration bacteria that can grow in this environment. The bacteria *Escherichia coli*, *Listeria monocytogenes*, Cryptosporidium, and Salmonella are pathogens that can be considered hazardous to human health and, depending on the type of juice, these germs must be eliminated in the processes aimed at controlling the effectiveness of disinfection treatments [1]. Suggested specifications for fruit juices in the Gulf region state the maximum permissible count with respect to total colony count of coliforms, yeast, and molds is 1 × 10^4^, 100, and 1 × 10^3^ CFU/mL, respectively [2].

Fruit juice treatments rely on thermal pasteurization and their natural acidity. However, the appearance of *E. coli* O157:H7 and other pathogens has raised concerns about the resistance of these pathogens to these treatments that require the total absence of these pathogens for microbiological safety [3,4]. The pH range for the majority of fruit juices, including orange juice, is in the acidic range (<4.5).

In the past, such acidic pH values would not have allowed for pathogen development. According to Foster [5], several *E. coli* strains showed substantially stronger tolerance to acidic pH and were able to adapt a number of acid stress survival strategies. *E. coli* multiplies at temperatures between 7 °C and 50 °C, the optimal temperature being 37 °C [6].

It is difficult to find alternatives to standard pasteurization because *E. coli* strains present a problem when processing orange juice, due in part to their resistance in acidic conditions and high temperatures [7].

Therefore, additional techniques that may inactivate the microorganisms can be used to reduce the undesirable effects of the process of thermal pasteurization (non-enzymatic browning, flavors, and loss of vitamins). Non-thermal techniques, such aspasteurization by hydrostatic high-pressure treatment (HPP), electric fields, and ultrasonic waves, are intriguing for this purpose [8]. Other options include ozone treatment [9], dynamic high pressure [10], pectin methyl esterase [11], clarification [12], cold atmospheric plasma [13], or a combination of such processes with low-temperature treatments [14].

To eliminate hazardous microorganisms from the food supply, non-thermal methods are often utilized in food processing. Unfortunately, these methods encounter difficulties throughout the inactivation process. The most cutting-edge non-thermal technology for guaranteeing the inactivation of germs whilst maintaining the quality of the fruit juices is the ultrasonic method (US). It is recognized as ecologically benign, energy-efficient, and minimal in physical and chemical dangers [15]. The ultrasound has a hydrodynamic effect (intracellular cavitations and microflocculation phenomena) and the generation of radicals that disturb the cell structure [16]. As a conservation method, using ultrasound only is not sufficient enough to destroy all microorganisms. Excessively, ultrasonic power could also damage the food’s flavors and nutritional value [17].

Therefore, a novel technique of heat treatment has been developed that uses a microwave instead of a direct heat source. Due to the decrease in processing time and costs, enhancement of product consistency and yields, development of a consistent microstructure, and protection of food from browning and surface crusting, it is extensively employed in the food industry [18].

So, combining microwaves and ultrasound may result in energy savings while maintaining the quality and efficacy of microbial inactivation [19,20,21,22]. Additionally, this method is very promising when used to decontaminate fruit juice because it can kill microbes at lower temperatures while keeping the juice’s qualities [23].

The main objective of optimization is to find the conditions that allow the best performance of a system to be obtained, which has been widely used as a conventional optimization method, based on the change of one variable, one factor at a time (OFAT). The primary flaws in this approach, however, are the unaccounted-for interactions between the variables and the absence of an explanation for how all of the components affect the response. Additionally, the study will need more tests under this approach, which will cost more and take longer [24]. Utilizing statistical multivariate methodologies, optimization research may be carried outto address this issue. The widely used multivariate statistical approach, the response surface method (RSM), was used to improve food processing [25].

In reviewing the literature, it was found that there was a lack of research on the combination of ultrasound and microwave applied and modeled with the response surface methodology (RSM) in mandarin juice. The objective of the present study was to optimize a combined microwave and ultrasonic process using response surface methodology to achieve the inactivation of *E. coli* in an orange juice drink.

## 2. Results and Discussion

### 2.1. Box–Behnken Analysis of E. coli Inactivation

The BBD was used in this method, which was carried out by RSM. The experiment’s findings revealed that all parameters, including microwave power (A), microwave treatment time (B), and ultrasound time (C), significantly affect the inactivation of *E. coli*. Throughout the test, these variables were kept constant and at the same values (OFAT). The values of levels and parameters for inputs are shown in Table 1 and the experimental findings of *E. coli* inactivation are shown in Table 2 after the BBD was modified to increase the amounts of these components and explore their interactions.

### 2.2. Performance and Fit of the RSM Model

ANOVA was used to assess the significance of the quadratic model, and the results are presented in Table 3.

With an F value of 29.63, this design is significant, and there is a 0.08% possibility that the F value is the result of noise. The model is composed of various terms, such as A (microwave power), B (Microwave treatment time), C (time of ultrasound), AB (microwave power vs. microwave treatment time), AC (microwave power vs. time of ultrasound), and A^2^ (microwave power^2^), and are significant because they have *p*-value less than 0.05. On the other hand, the factors BC (time of microwave vs. time of ultrasound), B^2^ (microwave treatment time^2^), and C^2^ (time of ultrasound^2^) are insignificant with *p*-values of more than 5%. With an F-value of 1.83, the lack of fit suggests that this is not noteworthy in comparison to the pure error. Noise has a 37.26% probability of producing a big lack-of-fit F-value; thus, a non-significant lack of fit is acceptable. The predicted values based on experimental data are estimated using the determination coefficient *R*^2^ value, and an *R*^2^ value of 0.9816 shows that the model is capable of carrying out its intended function. The model is generally considered to be appropriate in explaining the variability of the study results as the *R*^2^ value exceeds 0.75 [26]. 

An adjusted *R*^2^ value (0.9485) of the suggested model validates its accuracy. Taking into account the noise ratio, with the value of 16.9018 (Adeq Precision), it is considered as an appropriate response ratio and high accuracy. The model’s precision can be indicated, which should be greater than 4 [27]. The coefficient of variation (CV% = 21.57%) and Adequate Precision (Table 2) were also noted as indicators of its reliability. Compared with previous reports that compared the model’s accuracy with the predicted values, it was able to produce reproducible results [20,28].

**Figure 1:**
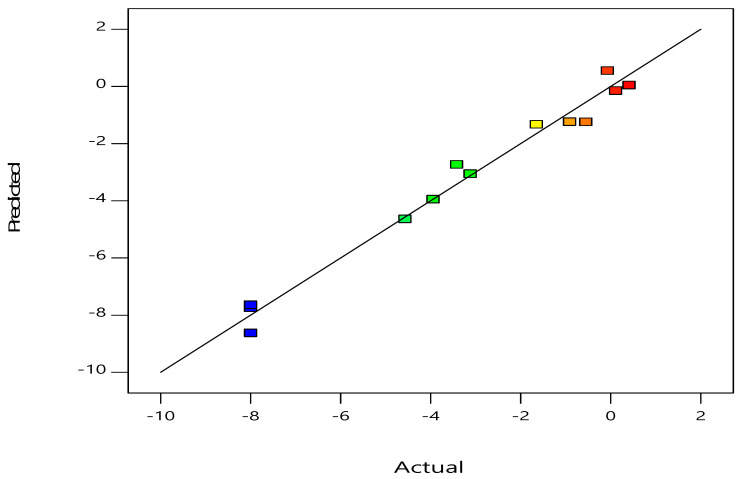
Relationship between the response surface model’s predicted and actual values for *E. coli* inactivation.

The findings from the expected vs. real values for the response surface method-assisted inactivation of *E. coli* are shown in Figure 1. A well-fitted model is suggested by the excellent connection between the expected and actual values of *E. coli* inactivation and the linear distribution. Values predicted from experimental data are estimated using the determination coefficient *R*^2^ value, in which an *R*^2^ value of 0.9816 indicates the ability of the model to perform the function for which it was designed. These results show this model is pretty realistic. Despite the slight differences between predicted and actual values [20], an *R*^2^ value of 0.9816% indicates that the design is able to reproduce the data accurately. The model’s statistical characteristics indicate that it is sufficiently accurate in terms of identifying the main effects of the components [29].

A final polynomial regression model equation for *E. coli* inactivation is based on the coded factors, and can be used to model the different factors that influence the inactivation process (Equation (1)).

(1) $$Log(N/N_{0})=-3.42-2.73A-1.98B-1.45C-1.40AB-0.94AC-0.023BC+0.978A^{2}+0.08B^{2}-0.58C^{2}$$

where *Log* (*N*/*N*_0_) is the response *E. coli* inactivation (CFU/mL), *A*; microwave power, *B*; time of microwave, and *C*; time of ultrasound. The positive and negative signs in the front of the design terms refer to the synergistic and antagonistic effects of the factors. A developmentally based design that uses coded factors is ideal because it can assist in identifying the most significant factors which will affect the response [30].

The study of the effects of various variables, such as microwave treatment time, ultrasonic time, and microwave power on the inactivation of *E. coli* in the orange juice drink indicated that the model with quadratic variance had a significant decrease in the amount of *E. coli*, and the non-significance of the lack of fit demonstrates the appropriate accuracy of the resulting model (Table 3).

### 2.3. Analysis of Interactions between Influencing Factors

Based on BBD regression analysis, three-dimensional interaction diagrams and response surface diagrams were employed to study relevant factor interactions and their impact on response.

**Figure 2:**
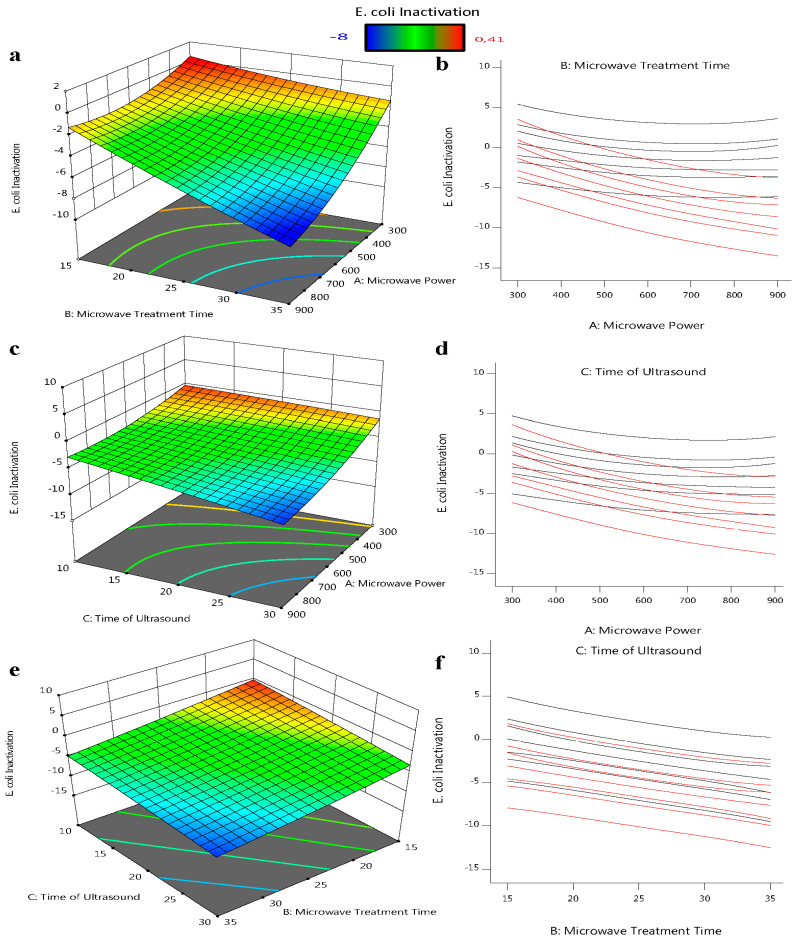
Three-dimensional response surface plots showing the influence of process factors on *E. coli* ATCC 25922: (**a**,**b**) the microwave treatment time and microwave power intersection; (**c**,**d**) the time of ultrasound and microwave power intersection; and (**e**,**f**) the time of ultrasound and microwave treatment time intersection for *E. coli* reduction.

Figure 2a,b shows that the increase inthe microwave time led to the decrease inthe bacterial load of *E. coli*, in which the power increase does not instigate the same response as a sudden increase in power. In the short term, increasing the microwave power does not directly affect the *E. coli* reduction rate trend; however, increasing the time for and extended amount of time leads to *E. coli* reduction. In the outcomes of Equation (2), the negative coefficient of microwave power multiplied by time can be seen. As a consequence, adding more independent variables leads to a higher negative value, which indicates a greater decrease in the quantity of *E. coli*. 

The microwaves’ chosen heating characteristics are what cause them to kill different kinds of bacteria and enzymes. The cell membrane collapses as the microwaves grow warmer than the surrounding liquid due to their dielectric characteristics [31].

In comparison to the time of microwave treatment, ultrasonic is more advantageous in reducing the *E. coli* in orange juice (Figure 2c,d); increasing the ultrasound exposure time contributes to an improved *E. coli* slope. Due to the formation of more sonic currents in the reactor as a result of longer ultrasound exposure, which increases the amount of ultrasonic waves that *E. coli* can absorb, longer ultrasound exposure contributes to a reduction in the amount of *E. coli* in the orange juice. This outcome is in line with research conducted on other fruit drinks. Response surface plots and interaction plots for the *E. coli* reduction model of the interaction between microwave power and ultrasound exposure duration were studied [32,33].

Figure 2e,f shows that the effect of a longer duration of ultrasound on cell viability does not change with time or temperature. However, with a *p*-value of 0.05, there is not a significant relationship between the variables of microwave time and ultrasound time. It was suggested that, at temperatures above a given threshold, the microbial inactivation rate for thermosonication does not increase compared to heat treatment. Raso et al. [34] further confirmed that the impact of inactivation was exclusively brought on by heat at temperatures over 58 °C. This may be because of the declumping effect, reduced cavitation activity, increased vapor pressure, and decreased surface tension that occur at high temperatures [35].

After optimization by RMS for the extraction of polyphenols in avocado skin by ultrasound and microwave-assisted methods, Trujillo-Mayol et al. [36] found that there is no interaction between microwave exposure time and ultrasound exposure time (*p*-value ˃ 0.05).

### 2.4. Validation of the Model

The objectives of the optimization process were to minimize energy consumption in order to reduce the *E. coli* content to zero. The data generated by the software Design Expert 11.6.0 was used to determine the optimal values for the various parameters of the model. The highest decrease in *E. coli* that was observed in the entire model served as the basis for setting variable level ranges, which were then put to the test in two trials. Table 4 displays the utilized value ranges. The validation process was performedunder the best conditions that the software design suggested. 

The data indicates that ultrasound uses much less energy than microwaves; thus, we picked the following figures as the best: 883.573 W for microwave power, 32.973 s for microwave duration, and 20.483 min for ultrasound time. Rounding each of the independent variables to the nearest whole number confirmed the values of *E. coli* ATCC 25,922 inactivation, which were set to zero. This ensures that the optimization method is the most reliable. As a result, the microwave power, microwave treatment time, and ultrasonic time were equal to 900 W, 33 s, and 20 min, respectively, and complete inactivation was achieved. This shows how precise the analysis and optimization processes are.

Based on FDA regulations, a 5-log decline was observed only during the 60 °C thermosonication process for *L. monocytogenes* [37], while the combined impact of ultrasound and microwave power could reach an acceptable inactivation level of about 8 log. It was observed that by increasing the temperature, the thermosonication process can be more efficient at inactivation of pathogenic bacteria [35]. Additionally, Anaya-Esparza et al. [38] reported that processing soursop nectar at 50 °C showed efficiency in decreasing the population of *E. coli* and *S. aureus* by around 5 log. Moreover, increased temperatures in the range of 50 °C and greater lead to protein denaturation, loss of membrane structural integrity and, ultimately, mortality of non-spore-forming pathogenic bacteria [39,40].

## 3. Materials and Methods

### 3.1. Conditions for Culture and Bacterial Strain

*Escherichia coli* ATCC 25,922 strains from the Institut Pasteur collection (Algiers, Algeria) were used in experiments. Prior to usage, this strain was kept on tryptone soy agar (TSA; Conda, Pronadisa, Spain) at 4 °C. Young culture bacteria were prepared using tryptone soy broth (TSB; Conda, Pronadisa, Spain). The colonies were then left suspended for 18 h at 37 °C. After that, they were spun at 4000 g for 15 min at 4 °C to separate them [41].

### 3.2. Inoculation of Orange Juice

The study was conducted using a commercial orange juice beverage with a pH of 3.27 and a 12° Brix (ROUIBA, Algeria). After filtering the liquid to remove the pulp, the previously prepared *E. coli* strain was added to it at a final concentration of 10^8^ CFU/mL [41].

### 3.3. Procedure of Treatment by Microwave and Ultrasound

The amount of inactivated *E. coli* was used to measure how well the waves killed microorganisms. 

*E. coli*’s inactivation in orange juice was tested using the method developed by Kernou, Belbahi, Amir, Bedjaoui, Kerdouche, Dairi, Aoun, and Madani [19]. They looked at the effects of microwave energies of 300 W, 600 W, and 900 W; microwave exposure times of 5 to 35 s; ultrasound exposure times of 10 to 30 min; and combinations of microwave and ultrasound on the inactivation of *E. coli* strains. This strain’s inactivation was optimized using the traditional OFAT approach (in which one parameter is changed while the others are maintained at specified values) and modeled using RSM.

### 3.4. Enumeration of Survival Cells

A sterile NaCl solution (0.9%) was used to serially dilute the orange juicebeverage radiated *E. coli* cell suspensions before spreading them over an Eosin Methylene Blue substrate (EMB; biolab, Hungary). The number of cells in the plates was counted after 24 h of incubation at 37 °C.

### 3.5. Optimal Inactivation of E. coli Using the RSM Method and the Box–Behnken Design Enumeration of Survival Cells

The Box–Behnken design (BBD) has been employed both for data analysis and optimization in order to get the best value [19]. This optimization’s parameters and levels were chosen using the traditional OFAT technique. In order to find out the initial range of *E. coli* inactivation variables, inactivation optimization focused on a single factor test that looked at A: the power of the microwave, B: the time the sample was exposed to the microwave, and C: the time the sample was exposed to the ultrasound (Table 5).

The influence factors and the levels of variability in the model were confirmed with the OFAT approach. Box–Behnken design (BBD, 3 variables) was performed with the help of 15 experiments (Table 5), which were conducted to fit the polynomial model. The process was performed to optimize the processes using data Design Expert 10^®^ software (Version 10.0.5.0., USA). The number of trials that were planned was determined according to Equation (2):

(2) $$N=2k\cdot (k-1)+C_{0}$$

*C*_0_ is the number of central points (3), and *k* is the number of factors. 

The trial data were looked at with the response surface model to find out how the variables and response factors were related [42]. Equation (3) was used to run a regression analysis in order to fit the second-order polynomial model. This technique was used to forecast the optimal conditions for *E. coli* inactivation.

(3) $$R=\beta_{0}+\sum_{i=1}^{n}\beta_{i}X_{i}+\sum_{i=1}^{n}\beta_{ii}{X_{i}}^{2}+\sum_{i=1}^{n}\sum_{i=1}^{n}\beta_{i}X_{i}X_{j}+\varepsilon$$

Here, *R* is the response surfaces; *β*_0_ is the constant (intercept) term; *β*_i_, *β*_ii_, and *β*_ij_ represent the linear, squared, and interaction coefficients, respectively; while *X_i_*, *X_i_*^2^, and *X_i_X_j_* are the linear variables, quadratic variables, and interaction term of the variables, respectively; and *ɛ* is the residual associated with the experiments (the prediction error is a statistical measure that reveals the difference between the predicted *R* values and the actual values and quantifies the random variability of the experiment). While keeping other elements constant, the surface plots were generated based on the values of two factors. Then, other interaction and linear models were incorporated to create the second-order polynomial model (Equation(4)) [43]. 

The determination coefficient (*R*^2^) value is used to gauge how well the model fits the data (Equation (4)). It is determined by bringing the model’s value closer to the value of 1.

(4) R2=1−∑i=1nRi−Rli2÷∑i=1nRi−R-2

where *n*, *R_i_*, *R_i_*, and $\overset{-}{R}$ are the number of measurements, the *i*-th observation value, the *i*-th prediction value, the number of trials, and the mean of response factors, respectively. Eliminating the prediction ability of a model can enhance its performance when the relevant item’s influence is significant. The **R*^2^* prediction is a statistical coefficient used to calculate the model’s prediction capabilities (Equation (5)).

(5) $${R^{2}}_{predict}=1-\left(1-R^{2}\right)\left[\left(n-1\right)÷\left(n-\left(k+1\right)\right)\right]$$

In the regression equation, “*n*” denotes the number of observations and “*k*” is the number of independent variables. The proximity of the *R*^2^ prediction to 1 indicates how well the model predicts the future. This technique involves rounding off the model’s tiny elements. The model’s ability to predict outcomes will increase with the development of the new RSM [42]. 

## 4. Conclusions

Microwave power, microwave treatment time, and ultrasonic exposure time are important factors to consider in order to reduce the amount of *E. coli* in orange juice. Due to the thermal effect, the amount of *E. coli* decreased when the microwave power was increased. The amount of *E. coli* decreased when the microwave power was increased, due to the thermal impact. Additionally, due to the increase in cavitation intensity caused by the ultrasound treatment, the number of *E. coli* decreased; as the duration of ultrasound exposure increases, the rate of drop of *E. coli* is initially considerable, but then eventually it starts to decrease. According to the ANOVA results, the interaction effect of microwave power and microwave time and the interaction of microwave power and ultrasound duration on the number of microbes was significant (*p* < 0.01). The drop rates of *E. coli* are initially considerable as the duration of ultrasound exposure of the orange juice drink increases, but they gradually decrease. Furthermore, the measured experimental results and the MSR-based optimization strategy were in very good agreement with each other. The microwave-ultrasound treatment was found to be a promising technology for the reduction and complete inactivation of the major pathogenic indicator microorganisms in an orange juice drink. In future studies, ultrasound and microwave treatment effects, such as sensory properties and aroma profile, should be examined.

## Figures and Tables

**Table 1:** Values of independent variables and corresponding proportions utilized in the RSM.

Independent Variable	Level		
	−1	0	1
Microwave power (Watt, A)	300	600	900
Microwave treatment time (second, B)	15	25	35
Time of ultrasound (minute, C)	10	20	30

**Table 2:** Plan for the experimental design and related BBD responses based on RSM for *E. coli* inactivation.

	Factor A	Factor B	Factor C	Response
Run	Microwave Power	Microwave Treatment Time	Time of Ultrasound	*E. coli* Inactivation
	W	s	min	Log (N/N_0_)
1	600	35	30	−8 ± 0
2	300	35	20	−0.037 ± 0.3
3	300	25	30	−0.370 ± 0
4	600	25	20	−3.94 ± 0
5	600	15	10	0.11 ± 0
6	300	25	10	0.07 ± 0
7	900	35	20	−8 ± 0
8	600	35	10	−4.47 ± 0
9	900	25	30	−8 ± 0
10	900	15	20	−1.8931 ± 0
11	600	25	20	−3.49 ± 0
12	600	15	30	−3.33 ± 0
13	300	15	20	0.48 ± 0
14	600	25	20	−2.83 ± 0
15	900	25	10	−3.8 ± 0

**Table 3:** Estimated polynomial quadratic model regression coefficients and analysis of variance (ANOVA) for the logarithmic *E. coli* decrease in orange juice.

Source	Sum of Squares	df	Mean Square	F-Value	*p*-Value	
Model	124.44	9	13.83	29.63	0.0008	Significant
A-microwave power	59.60	1	59.60	127.71	<0.0001	
B-microwave treatment time	31.50	1	31.50	67.49	0.0004	
C-time of ultrasound	16.85	1	16.85	36.10	0.0018	
AB	7.81	1	7.81	16.74	0.0094	
AC	3.53	1	3.53	7.57	0.0402	
BC	0.0020	1	0.0020	0.0043	0.9500	
A^2^	3.53	1	3.53	7.56	0.0403	
B^2^	0.0236	1	0.0236	0.0506	0.8309	
C^2^	1.25	1	1.25	2.68	0.1623	
Residual	2.33	5	0.4667			
Lack of Fit	1.71	3	0.5700	1.83	0.3726	Not significant
Pure Error	0.6234	2	0.3117			
**Cor Total** *R*^2^ Adjusted *R*^2^ AdeqPrecision C.V. %	126.77	14		0.9816 0.9485 16.9018 21.57		

**df** Degree of Freedom.

**Table 4:** Validation of the RSM-optimized quadratic model for *E. coli* inactivation by microwave and ultrasound in an orange juice beverage.

Solution Number	Microwave	Microwave Treatment Time	Time of Ultrasound	*E. coli* Inactivation Actual	*E. coli* Inactivation Predicted	Std. Err	Desirability
	W	S	min	Log (N/N_0_)	Log (N/N_0_)		
1	600	35	30	−8.000	−8.000	0.553	1
2	900	33	20	−8.000	−8.000	0.487	1

**Table 5:** Independent factors and levels of variation in the Box–Behnken design.

Study Type	Response Surface	Subtype			Randomized	
Design type	Box–Behnken		Runs 15			
Design mode	Quadratic		No blocks			
Factor	Name	Units	Type	Minimum	Maximum	Mean
*A*	Microwave	Watt	Numeric	300	900	600
*B*	Microwave treatment time	Second	Numeric	15	35	25
*C*	Time of ultrasound	Minute	Numeric	10	30	20
Response	Name	Units	Obs	Analysis		
*R*	Inactivation	-	15	Polynomial		

## Data Availability

The data are available from the corresponding author.

## References

[1] Lima Tribst A.A., de Souza Sant’Ana A., de Massaguer P.R. (2009). Microbiological quality and safety of fruit juices—Past, present and future perspectives. Crit. Rev. Microbiol..

[2] Standard G. (2000). Microbiological Criteria for Food Stuffs-Part 1.

[3] Yap M., Ercolini D., Álvarez-Ordóñez A., O’Toole P.W., O’Sullivan O., Cotter P.D. (2022). Next-generation food research: Use of meta-omic approaches for characterizing microbial communities along the food chain. Annu. Rev. Food Sci. Technol..

[4] Badenhorst A.B. (2020). The Risk of Pathogenic Microbiological Contamination of South African Fresh Fruit for the Export and Local Market. Ph.D. Thesis.

[5] Foster J.W. (2001). Acid stress responses of Salmonella and *E. coli*: Survival mechanisms, regulation, and implications for pathogenesis. J. Microbiol..

[6] Pal Roy M., Mazumdar D., Dutta S., Saha S.P., Ghosh S. (2016). Cloning and expression of phytase appA gene from Shigella sp. CD2 in Pichia pastoris and comparison of properties with recombinant enzyme expressed in *E. coli*. PLoS ONE.

[7] Wang Z., Fang Y., Zhi S., Simpson D.J., Gill A., McMullen L.M., Neumann N.F., Gänzle M.G. (2020). The locus of heat resistance confers resistance to chlorine and other oxidizing chemicals in *Escherichia coli*. Appl. Environ. Microbiol..

[8] Toepfl S., Heinz V., Knorr D. (2007). High intensity pulsed electric fields applied for food preservation. Chem. Eng. Process. Process Intensif..

[9] Patil S., Bourke P., Frias J.M., Tiwari B., Cullen P. (2009). Inactivation of *Escherichia coli* in orange juice using ozone. Innov. Food Sci. Emerg. Technol..

[10] Tahiri I., Makhlouf J., Paquin P., Fliss I. (2006). Inactivation of food spoilage bacteria and *Escherichia coli* O157: H7 in phosphate buffer and orange juice using dynamic high pressure. Food Res. Int..

[11] Torres E., González-M G., Klotz B., Rodrigo D. (2016). Effects of high hydrostatic pressure and temperature increase on *Escherichia coli* spp. and pectin methyl esterase inactivation in orange juice. Food Sci. Technol. Int..

[12] Anvarian A.H., Smith M.P., Overton T.W. (2016). The effects of orange juice clarification on the physiology of *Escherichia coli*; growth-based and flow cytometric analysis. Int. J. Food Microbiol..

[13] Dasan B.G., Boyaci I.H. (2018). Effect of cold atmospheric plasma on inactivation of *Escherichia coli* and physicochemical properties of apple, orange, tomato juices, and sour cherry nectar. Food Bioprocess Technol..

[14] Rifna E., Singh S.K., Chakraborty S., Dwivedi M. (2019). Effect of thermal and non-thermal techniques for microbial safety in food powder: Recent advances. Food Res. Int..

[15] Mohideen F.W., Solval K.M., Li J., Zhang J., Chouljenko A., Chotiko A., Prudente A.D., Bankston J.D., Sathivel S. (2015). Effect of continuous ultra-sonication on microbial counts and physico-chemical properties of blueberry (*Vaccinium corymbosum*) juice. LWT.

[16] Tiwari B., Muthukumarappan K., O’Donnell C., Cullen P. (2008). Effects of sonication on the kinetics of orange juice quality parameters. J. Agric. Food Chem..

[17] Ferrario M., Alzamora S.M., Guerrero S. (2015). Study of the inactivation of spoilage microorganisms in apple juice by pulsed light and ultrasound. Food Microbiol..

[18] Huang Y., Sheng J., Yang F., Hu Q. (2007). Effect of enzyme inactivation by microwave and oven heating on preservation quality of green tea. J. Food Eng..

[19] Kernou O.N., Belbahi A., Amir A., Bedjaoui K., Kerdouche K., Dairi S., Aoun O., Madani K. (2021). Effect of sonication on microwave inactivation of *Escherichia coli* in an orange juice beverage. Food Process Eng..

[20] Rostami S., Behruzian M., Samani B.H., Lorigooini Z., Hosseinabadi T., Zareiforoush H., Behruzian A. (2018). Study of combined ultrasound-microwave effect on chemical compositions and *E. Coli* count of rose aromatic water. Iran. J. Pharm. Res..

[21] Demirok N.T., Yıkmış S. (2022). Combined Effect of Ultrasound and Microwave Power in Tangerine Juice Processing: Bioactive Compounds, Amino Acids, Minerals, and Pathogens. Processes.

[22] Das M.J., Das A.J., Chakraborty S., Baishya P., Ramteke A., Deka S.C. (2020). Effects of microwave combined with ultrasound treatment on the pasteurization and nutritional properties of bottle gourd (Lagenaria siceraria) juice. J. Food Process. Preserv..

[23] Bhat R., Kamaruddin N.S.B.C., Min-Tze L., Karim A. (2011). Sonication improves kasturi lime (*Citrus microcarpa*) juice quality. Ultrason. Sonochem..

[24] Bezerra M.A., Santelli R.E., Oliveira E.P., Villar L.S., Escaleira L.A. (2008). Response surface methodology (RSM) as a tool for optimization in analytical chemistry. Talanta.

[25] Baş D., Boyacı I.H. (2007). Modeling and optimization I: Usability of response surface methodology. J. Food Eng..

[26] Omar W., Nordin N., Mohamed M., Amin N. (2009). A two-step biodiesel production from waste cooking oil: Optimization of pre-treatment step. Appl. Sci..

[27] Shojaei S., Shojaei S. (2017). Experimental design and modeling of removal of Acid Green 25 dye by nanoscale zero-valent iron. EuroMediterr. J. Environ. Integr..

[28] Hosseinzadeh Samani B., Khoshtaghaza M., Minaee S. (2018). Modeling the simultaneous effects of microwave and ultrasound treatments on sour cherry juice using response surface methodology. J. Agric. Sci. Technol..

[29] Banu A., Ali M.Y., Rahman M.A., Konneh M. (2020). Stability of micro dry wire EDM: OFAT and DOE method. Int. J. Adv. Manuf. Technol..

[30] Montgomery D.C. (2017). Design and Analysis of Experiments.

[31] Tajchakavit S., Ramaswamy H., Fustier P. (1998). Enhanced destruction of spoilage microorganisms in apple juice during continuous flow microwave heating. Food Res. Int..

[32] Wu J., Gamage T., Vilkhu K., Simons L., Mawson R. (2008). Effect of thermosonication on quality improvement of tomato juice. Innov. Food Sci. Emerg. Technol..

[33] Samani B.H., Khoshtaghaza M.H., Lorigooini Z., Minaei S., Zareiforoush H. (2015). Analysis of the combinative effect of ultrasound and microwave power on *Saccharomyces cerevisiae* in orange juice processing. Innov. Food Sci. Emerg. Technol..

[34] Raso J., Pagan R., Condon S., Sala F. (1998). Influence of temperature and pressure on the lethality of ultrasound. Appl. Environ. Microbiol..

[35] Ugarte-Romero E., Feng H., Martin S.E. (2007). Inactivation of Shigella boydii 18 IDPH and Listeria monocytogenes Scott A with power ultrasound at different acoustic energy densities and temperatures. J. Food Sci. Technol..

[36] Trujillo-Mayol I., Céspedes-Acuña C., Silva F.L., Alarcón-Enos J. (2019). Improvement of the polyphenol extraction from avocado peel by assisted ultrasound and microwaves. J. Food Process Eng..

[37] Jafarpour D., Hashemi S.M.B., Mousavifard M. (2022). Inactivation kinetics of pathogenic bacteria in persimmon using the combination of thermosonication and formic acid. Food Sci. Technol. Int..

[38] Anaya-Esparza L.M., Méndez-Robles M.D., Sayago-Ayerdi S.G., García-Magaña M.d.L., Ramírez-Mares M.V., Sánchez-Burgos J.A., Montalvo-González E. (2017). Effect of thermosonication on pathogenic bacteria, quality attributes and stability of soursop nectar during cold storage. CyTA-J. Food.

[39] Parreiras P.M., Nogueira J.A.V., da Cunha L.R., Passos M.C., Gomes N.R., Breguez G.S., Falco T.S., Bearzoti E., Menezes C.C. (2020). Effect of thermosonication on microorganisms, the antioxidant activity and the retinol level of human milk. Food Control.

[40] Russell A. (2003). Lethal effects of heat on bacterial physiology and structure. Sci. Prog..

[41] Cabassi C.S., Falanga G., Romani A. (2017). Disinfectant and Antimicrobial Compositions, in Particular for the Veterinary Field. U.S. Patent.

[42] Xie Y., Hu P., Zhu N., Lei F., Xing L., Xu L. (2020). Collaborative optimization of ground source heat pump-radiant ceiling air conditioning system based on response surface method and NSGA-II. Renew. Energy.

[43] Srivastava A., Singh V., Haque S., Pandey S., Mishra M., Jawed A., Shukla P., Singh P., Tripathi C. (2018). Response surface methodology-genetic algorithm based medium optimization, purification, and characterization of cholesterol oxidase from *Streptomyces rimosus*. Sci. Rep..

